# On the Obscure Mental Disorders of Criminals

**Published:** 1859-01-01

**Authors:** 


### Art. IV.—ON THE OBSCURE MENTAL DISORDERS OF CRIMINALS.

The mental disorders of criminals are of singular interest, more particularly those obscure affections which may be arranged as a transition series between positive criminality on the one hand, and criminality as the result of manifest insanity on the other. Cases of this kind are distinguished, perhaps, more by the anomalous mode in which the vicious or criminal propensities are manifested than in any other fashion; and their importance (as yet, perhaps, not sufficiently recognised) can scarcely be exaggerated; for, from the circumstances under which they are observed, they offer to the psycopathist an excellent opportunity for the careful and systematic study of many of those slight shadings off of the healthy into the morbid action of the brain, which are all-important in mental pathology and therapeutics. These cases, moreover, are equally important to the medical jurist; for from them, he may, with the greatest probability, hope to obtain many satisfactory aids in the diagnosis of certain obscure forms of insanity accompanied by criminal acts, for the want of which he is not unfrequently placed at disadvantage in a court of law.

A prisoner confined in one of our large prisons is so situated that he may be observed steadily and systematically, and the conditions under which he is placed, and their influence upon him, may be fully ascertained and accurately estimated. The evidence, therefore, which would be obtained regarding obscure mental affections under these circumstances might be expected to have peculiar value; and in its bearing upon the legal doctrines of the responsibility of the insane, it would doubtless have great weight, removed as it would be from the refracting medium of the procedure of a court of law. No evidence probably would more certainly and quickly operate with both the public and the bar in inducing them to admit the justice of the proposition, that when positive indications of the existence of a morbid state of the mind are ascertained to exist in a prisoner at the bar, he ought to be dealt with after a different fashion to the ordinary criminal. Punishment in such cases is not only useless, but it is commonly harmful; and if hopeless insanity be not determined by it, it most probably will aggravate the vicious disposition of the prisoner, and at the termination of his period of imprisonment he will forthwith be guilty of other and more serious criminal acts.

Prisoners of the class referred to require to be treated as lunatics and not as criminals, and their confinement should have for object the testing of their true state of mind, and the adoption of such means for relief as may be deemed necessary; while their release should be made dependent, in a great measure, upon the condition of the mind at the time.

In illustration of the foregoing remarks, and as a valuable contribution to mental pathology, we may quote several cases of the more obscure mental disorders of criminals which are recorded in the recently published Reports of the Directors of Convict Prisons in England, and also in Ireland.

Mr. Bradley, the able medical officer of the Pentonville prison, reports the following cases:—

"W. G., aged 34, a labourer from Westmoreland, was tried at Worcester, on the 6th of March, 1854, and sentenced to four years' penal servitude, for a burglary. He was first confined in the city of Worcester Gaol, for about five months, and at his departure the governor gave him a character to the following effect:—His conduct was most refractory; he twice attempted to break out of his cell: he smashed the windows; *many times threatened the life of the governor and surgeon;* his ordinary language was too disgusting for repetition; and, in short, he was the 'vilest brute' he ever had in custody during a governorship of 35 years. From Worcester, the prisoner was sent to Millbank, where he remained but a few weeks, and was thence passed on to Pentonville. Here he was confined for upwards of a year, during the whole of which period, his conduct was marked by insubordination. He disturbed the prison, attempted an escape, *threatened the lives of the officers*, and to carry out his threats *devised several weapons of very ingenious construction.* When the term of separate confinement had expired, he was removed to Portland, where, as may be learned from his papers, he was, on account of highly mutinous and insubordinate conduct, after about three months' detention, removed to Millbank Prison, to be placed in the penal class. When he had been about five months in the last-named prison *he committed a murderous assault upon a warder*, for which offence he was sent to Newgate, tried at the Central Criminal Court, and sentenced to transportation for life. In pursuance of this sentence, he was, on the 14th April, 1856, sent to Pentonville for the second time. On entering the prison, and while in that courtyard awaiting his formal reception, *he threatened repeatedly that he would murder an officer* before the expiration of his imprisonment. His subsequent conduct was very similar to what it was during his former imprisonment here. He opposed himself to the rules, and murmured at the dietary, which he asserted was insufficient to maintain his strength. Sometimes he pretended to be too weak to leave his bed, or that he was actually at the point of death; at other times he maintained that immediate death was preferable to the completion of his sentence, and begged that he might be put to death. All this time his bodily health was good, and he retained his natural stoutness. The character originally given him by the Governor of Worcester Gaol was, to a certain extent, confirmed; for he appeared to be lazy and ill-conducted, morose, bloody-minded, and an impostor. His term of imprisonment in separation having expired, he would have been passed on to one of the public works prisons, but that the whole tenor of his prison life rendered it probable that insanity in a latent or unrecognised form was present, although neither incoherence nor delusion was evident. It was therefore considered advisable to recommend his removal to Dartmoor, where he would be under medical observation while his mental condition continued to be in an unsatisfactory state, and where the discipline would be better suited to his case than that of the public works. Before the necessary warrant arrived, he one day preferred a request to be allowed some indulgences in addition to his diet, and when this was refused as unreasonable, *he savagely attacked the medical officer, and stabbed him* with a weapon he had previously constructed for the purpose, which he had kept concealed in his coat sleeve, wounding also two of the warders who came to the rescue. When spoken to shortly afterwards on the serious nature of his offence, he expressed no contrition, but, on the contrary, regretted that he had not 'killed the doctor,' as he had intended, alleging that for some time past his food had been 'powdered' or poisoned by the medical officer's orders. As this was manifestly an insane delusion, the prisoner was placed in the infirmary, where he was visited by an '*expert*' in insanity, who pronounced him to be a proper subject for a lunatic asylum. In accordance with this view of the case, in which I fully concurred, the prisoner was, on the 9th of April, removed to Bethlem Hospital, whence, after a detention of somewhat less than five months, he was, on the 29th of August, sent back to Pentonville. *With murderous threats* in his mouth, lavishing abuse upon the establishment he had just left, and persisting in the story of the 'powdered' food, his mental condition on readmission was but little altered since I last saw him. As I was convinced from long-continued observation of the case that the prisoner was still the subject of mental affection, and unfit, therefore, for the discipline of the prison, he was recommended for removal to Dartmoor, whither he was sent on the 7th of September, accompanied by a brief statement, in order to put the authorities of that prison on their guard concerning him. From Dartmoor I am informed he has been removed on account of bad conduct to Millbank, where I believe he at present remains, and in the penal class.

"I am fully persuaded that the above case should be regarded as one of insanity, with homicidal tendency, an example of the *monomanie homicide* described by Esquirol, and as such to be rather a subject for treatment in a properly-constructed hospital for lunatics, than for the discipline of a prison. . . . .

"W. W., aged 22, a carter and a reputed thief, was convicted at the Bolton Sessions, October 6th, 1853, of larceny, after a previous conviction, and was sentenced to six years' penal servitude. It appeared from the Caption Papers that he was first confined for about six months in the New Bailey Prison, where he was 'sullen,' 'idle,' 'insubordinate,' and was whipped for refusing to work. Thence he was sent to Wakefield Prison, where his conduct during an imprisonment of nine months was 'bad.' Thence to Portland, where he was detained between 11 and 12 months, and where he obtained the following character from the governor: '*very bad*,' '*a most insubordinate and idle prisoner*,' '*I fear incorrigible*.' From Portland he was removed to Millbank. There he remained 15 months, during which period his general conduct was 'bad,' and he was flogged for insubordination. From Millbank he was passed on to Portsmouth. He remained in the latter prison only 26 days, and was then, on account of refusal to work and continuous insubordination, removed (on the 20th January last) to Pentonville, to undergo a third period of probation in separate confinement. During his imprisonment here his conduct was very similar to what it appears to have been in other prisons. He was generally idle and insubordinate. At times he was violent, smashing the windows, and *threatening the lives of the officers*. Although no delusion was manifested, yet the silly laugh, the motiveless misconduct, and other features of the case, sufficiently indicated the existence of weakness or unsoundness of mind, and the necessity for special treatment. The prisoner was placed in the infirmary, and put to associated labour. Subsequently (on the 22nd of April) he was removed to Dartmoor, as an unfit subject for separate confinement. He was visited and examined by Dr. * * * *, who gave an opinion to the effect that the mental condition of the prisoner was such as to excite a grave suspicion as to his responsibility, although the symptoms were not sufficiently pronounced to justify a removal to a lunatic asylum.

"T. K., aged 17, convicted on the 3rd of November, 1856, of burglary, and sentenced to six years' penal servitude, was received at Pentonville, 6th April, 1857. He was a somewhat weak-minded lad who, when he had been here about seven months, exhibited considerable excitement, and gave way to paroxysms of ungovernable violence. Under suitable moral treatment in the infirmary, where he received special attention from the schoolmaster, he became orderly and tractable, and at present encourages a hope of amendment and recovery.

"H. W., a prisoner of sullen disposition, when he had been exactly a month in the prison, contrived to open a vein in his arm with a steel pen, avowedly with a suicidal intention. A second time he resorted to a similar proceeding; and when means were adopted to prevent him from inflicting injuries of this kind on himself, he threatened to starve himself, and for several days refused food, until at length, finding that nourishment was about to be administered by means of the stomach-pump, his resolution forsook him, and he took his meals in a natural manner. He was treated for some time in the infirmary in association, and subsequently was removed to Dartmoor as an unfit subject for separate confinement.

"R. D., received on the 17th of September from Dartmoor, was removed to Millbank as an unfit subject for the discipline of this prison, at the first convenient opportunity after his reception. The prisoner, aged 24, had been a private in the Royal Marines. He was tried on the 28th of September, 1854, by a general court-martial, and sentenced to 14 years' transportation, for striking his serjeant. In pursuance of his sentence he was first sent to Maidstone Gaol. His conduct there was violent and disorderly, and after a detention of four months he was pronounced to be mad, and was removed to Bethlem. He was confined as a lunatic there and at Fisherton for about a year and three quarters, and was then re-conveyed to Maidstone Gaol. He remained two months in Maidstone, was again pronounced to be mad, and was again placed in Bethlem Hospital. After the lapse of five weeks he was discharged thence as sane, and removed to Millbank. At Millbank he was detained about six weeks. His conduct during that period was 'bad,' and he is said to have feigned an attempt at suicide. He was then removed to Portland, where his conduct for two months was 'very bad.' He attempted suicide, and was sent to Dartmoor. At Dartmoor he used threatening language, violently assaulted the officers, and was then, after three months' detention, removed to Pentonville, to undergo a period of probation in separate confinement.

"Such is a bare outline of this case, obtained from the prisoner's papers. From the time of his reception into Pentonville until his removal he conducted himself in so frantic a manner as to raise a grave suspicion of his sanity, independent of his previous history, which I think sufficiently shows that he was at all events an unfit subject for the discipline of separate confinement."

Upon three of these cases Mr. Bradley makes the following pertinent remarks:—

"The above case, that of W. G., and the two cases immediately following it, possess many features in common, and differ from each other but little, I believe, except in degree. They illustrate a peculiar class of prisoners received into Pentonville and the convict prisons at the present time. Prisoners of the class referred to are characterized by inveterate idleness, obstinacy, and insubordination, by gross and apparently motiveless misconduct. They are at intervals violent, and smash everything within reach. They assault officers, disturb the prison by shouting, and set all order at defiance. Some are also intractable malingerers; others threaten or attempt suicide. Such men occupy as it were a neutral territory between crime and insanity, oscillating from one to the other, until at length in some cases incoherence or delusion becomes apparent, the mental equilibrium is perceived to be lost, and they fall obviously into the domain of insanity; in other cases the mental condition continues doubtful, and in the prisons they are as often regarded as 'cracked' or crazy as they are in the lunatic asylums as criminals and impostors. On these the authorized prison punishments are found to be worse than useless, and the existing systems of discipline, whether 'separate' or associated, appear to be productive of little benefit. To deal effectively with them before actual insanity is established, a special and peculiar discipline is needed; for, so far as my experience extends, separate confinement is not attended by any good result; and as they are frequently sent back to us from the public works, it would seem that the discipline there is equally fruitless."

Dr. Maurice Corr, the medical superintendent of the Philipstown Government Prison (Ireland), reports that during the last eighteen months he had been under the necessity of recommending that fourteen convicts should be transferred to a lunatic asylum. Several of the patients were entitled to their discharge at the time of their removal to the Central Asylum, and of these cases we are told that they had not expressed any desire for liberty, notwithstanding a lengthened imprisonment and frequent punishment; neither had they shown any regret under severe privations, nor any inclination to amend.

The following cases are quoted from the Appendix to Dr. Corr's Report:—

"J. H., sentenced to ten years' transportation, October, 1850. Transferred to Central Asylum, October, 1856. Entitled to discharge, on commuted sentence, October, 1856.

"*Noted on committal sheet to Philipstown.*—'Violent, mischievous, and very easily excited.' *Character at Philipstown.*—Extremely eccentric. *Conduct at Philipstown.*—He was invariably insolent to his superiors; disobedient to the utmost; addicted to inordinate fits of laughter, particularly during Divine Service, for which reason he was, at length, kept from attending chapel; repeatedly violent to officers and prisoners, rendering it necessary to retain him in almost continuous separation, which did not appear to annoy him in the least. After constant and careful surveillance I could not arrive at other conclusion than that this prisoner was of unsound mind, that he offered no hope of improvement while retained in separation, and that it would be extremely dangerous to place him in association."

"J. L., sentenced to seven years' transportation, October, 1852. Transmitted to Central Asylum, January, 1857. Entitled to discharge on commuted sentence, October, 1856. Re-committed to Philipstown from Central Asylum, December, 1857.

"*Character at Philipstown.*—Treacherous, excitable to dangerous violence, insolent, disobedient, and not to be trusted in association. *Conduct at Philipstown.*—Attempted to commit suicide, when detected in a plan to assault an officer; assaulted a warder with a trussel, another with a stone, the deputy-governor with a bucket—all these attacks being most treacherous; severely wounded one of a class of prisoners, at whom he flung a brick, and, although unobserved in the act, he voluntarily admitted it; stealthily, at night, endeavoured to burn his clothes by placing them in the stove fire; subject to outbreaks of passion approaching to frenzy, during which it was absolutely necessary, for his self-preservation, to place and retain him under restraint. His demeanour and conversation were remarkably strange in hospital, where he was, on two occasions, treated for violent pain in his head. In general, he obstinately refused to attend Divine Service. Such were some of the grounds on which I concluded that 'J. L. *laboured under*

*dangerous mental aberration, with periodical fits of insanity;*' a conclusion not unnatural when dealing with a prisoner (if his conduct had been exemplary) entitled to discharge, who patiently endured repeated separations, personal restraint, low diet, &c., and who never expressed a wish or desire for his liberty. This prisoner, in two days after his arrival at Philipstown from the Central Asylum, made use of violent and obscene language to a warder, for which misconduct he was placed in a punishment cell where he remains up to this date, 11th January."

"J. R., sentenced to seven years' transportation, July, 1853. Transferred to Central Asylum, September, 1857. Entitled to discharge on commuted sentence, July, 1857.

"*Character at Philipstown.*—Treacherous, violent, dangerous; showed unmistakable signs of approaching fits, such as doggedness, refusal of food, eccentric conduct at exercise, hatred of those around him, &c. *Conduct at Philipstown.*—Repeated outbreaks of treacherous violence; broke windows, buckets, and articles within reach; tore up bed clothes and wearing apparel; made nuisance in cell, daubed the walls with it; assaulted officers; blasphemed loudly for hours; attempted to break out of cell; wore his clothes in a peculiar way; continually picked and scratched his private parts; prone to destroy walls and furniture. During lucid intervals spoke most rationally, so as to deceive strangers as to his real state, displaying considerable powers of reasoning; evincing desire for books, chiefly scriptural, which after a time he suddenly tore to pieces; invariably declined clerical advice; *was perfectly indifferent regarding his liberty, though well aware that his detention resulted from his own misconduct, and recoiled at mention of being restored to his friends.* Trials at association failed, owing to his frequent treacherous assaults on officers and prisoners. He was kept in separate confinement *for twelve* months up to his removal to Central Asylum. I considered him to be a dangerous lunatic."

"J. D., or H., sentenced to ten years' transportation, June, 1851. Transferred to Central Asylum, August, 1856. Entitled to discharge on commuted sentence, June, 1857.

"*Marked on committal sheet to Philipstown.*—'Supposed malingerer.' *Character at Philipstown.*—Remarkably silent, melancholy, morose, very quarrelsome when roused, even by speaking to him. *Conduct at Philipstown.* Had the habit of sitting for continuous hours on the sill of his cell window, shouting incessantly and becoming most violent when any attempt was made to remove him; would suddenly scatter and destroy his food, and, if remonstrated with by the warders, became dangerously excited, making desperate attempts to assault the parties present, succeeding, on one such occasion, in wounding the chief warder. After matured observation I considered it extremely dangerous to permit this prisoner into association. I looked upon him to be dangerously insane at intervals. I foresaw no prospect of amendment in separation.

"The inspectors of Lunatic Asylums state, in their Report for 1855–7 that 'D. is passionate, of very limited understanding; is of unsound mind, is a case for detention.'"

"*W. H.*, sentenced to seven years' transportation, July, 1853.

Entitled to discharge on commuted sentence, July, 1857. Remains at Philipstown.

"*Character noted on committal sheet to Philipstown.*—'Repeatedly punished for insolence—for assaulting officers—afterwards considered to be of unsound mind.' *Character at Philipstown.*—While tranquil he continually laughs like a fool; gives incoherent answers; appears to have extraordinary ideas about religion; retained from attending Divine Service, in consequence of his unruly conduct during it; subject to frequent fits of extreme violence. *Conduct at Philipstown.*—Assaulted officers; destroyed windows, bed-furniture, and body-clothes; passed urine and excrement on floor of cell. It has been found necessary to retain this prisoner in uninterrupted separation during the last twelve months, *no appearance of amendment being observed*, while he repeatedly offered such violence as to require personal restraint, reduction of diet, &c.—treatment endured without a remark and with evident indifference. He never alludes to his protracted separation; to his loss of commutation of sentence; nor speaks of his liberty. I certified that he was of unsound mind, subject to frequent paroxysms of dangerous lunacy."

"T. M'L., sentenced to four years' penal servitude, January, 1855. Transferred to Central Asylum, September, 1856.

"*Noted on committal sheet to Philipstown.*—'Is extremely violent when excited; is at all times most insolent; has been for a long time in separation, under medical surveillance.' *Character at Philipstown.*—He was exceedingly quarrelsome and easily excited to violence when checked; was an habitual blasphemer, an everlasting talker. *His conduct at Philipstown* brought on him repeated punishments with continued separation, of which he seemed perfectly regardless. His habits, previously to his conviction, were extremely intemperate, and led to frequent punishments for drunkenness. After continuous close observation, while he was in and out of separation, I concluded that he could not, with safety, be left in association; that he was subject to frequent aberrations of mind, which rendered him irresponsible for his acts; that, therefore, he should be deprived of opportunity to commit assaults, even though his insane paroxysms were interrupted by lucid intervals—a fact that, under all the circumstances of his case, including his former intemperance, induced me to recommend his transfer to the Asylum, as presenting favourable prospects of recovery."

Dr. Corr further informs us that the Philipstown Prison—

"Contains, under medical observation, a class rather numerous, of weak-minded, passionate, irresponsible convicts, who, without presenting decided symptoms of lunacy, are absolutely unfit for associated prisons, by reason of their dangerous propensities, easily-excited violence, and constant retention of officers and prisoners in fear of their temper and irregularities. Such a class is entirely unsuited to undergo the mildest form of discipline, against which each, after his own fashion, offers resistance, more or less violent. The sane portion of the prison population act in various ways towards men so affected, and help thereby to weaken discipline. The consequences are inevitable—the class referred to must be locked up in separation, and thus a case, in its incipient stage *quite curable*, steals along, under such treatment, into *confirmed lunacy*."

Mr. Awly Banon, the medical officer of the Grangegorman Female Prison, in his report upon the sanitary and medical condition of that prison, refers to a series of cases of doubtful lunacy occurring among the female convicts, in the following words :—

"In my report for the year 1856, there occurs the following paragraph :—'There is a class amongst the convicts with whom I find it very difficult to deal; I allude to those who, though they cannot be pronounced actually insane, are of such defective mental organization as to render them, in my opinion, not wholly responsible for the violence and excitement they too often exhibit. Of this class there are about six at present in the prison, two or three of whom require restraint and occasional separation.' My attention during the year has been particularly directed to some of these women, whose conduct has given considerable trouble. Two of them, especially J. D. and E. P., were very frequently brought before the Directors for violent and outrageous conduct, the former frequently assaulting her fellow-prisoners, and even the matrons, without any apparent motive or cause whatever. This woman had been sent up from Cork Prison on the 12th May, 1857, to Newgate Prison, where she remained, until transferred to Grangegorman on the 18th July, 1857. Her conduct in Cork, Newgate, and Grangegorman, has been throughout bad; kindness or punishment being equally unavailing in correcting her in the slightest degree. The other prisoner, E. P., has been in Grangegorman for nearly three years, and she also has very often been equally violent, breaking the glass in her cell, and giving way to fits of passion without the slightest reason, over which she seems not to have the least control, but soon afterwards expressing her contrition. Under these circumstances, I have been frequently referred to, as to whether these women are proper subjects for punishment, or whether I consider them unaccountable for their conduct from defective intellect, and therefore fit subjects for transfer to the Criminal Lunatic Asylum. Not having been able, on frequent and careful examination, as yet to detect any symptoms of insanity, other than those already mentioned, I have, up to this time, declined to certify for their transfer, but continue to have them under constant observation, with a view to their ultimate disposal. Their punishments are generally modified, being principally confined to restraint, by the wearing of the new jacket for a short period; their physical strength not being such as to justify, for any lengthened period, their being placed on bread and water. It is but natural to suppose, that amongst so many, we must expect to meet with some few cases calculated to give embarrassment, and I cannot now give any other suggestions for the better management of these women, than to continue to keep them under constant surveillance and medical treatment when necessary; and should either of them exhibit further symptoms of insanity, to have her at once transferred to the lunatic asylum. One or two others amongst the convicts exhibit symptoms of weak intellect, but not one of the troublesome character of those above mentioned. One of them, M. A., whose commuted term of imprisonment expired, refuses to tell who her friends are, and is frequently found muttering to herself, and showing, by other symptoms, that she is decidedly of unsound mind. I think the best course, in her case, would be to send her to the district Lunatic Asylum on being discharged from prison, should her friends not be forthcoming."

In what manner prisoners of doubtful sanity should be dealt with becomes a question of very considerable importance. It is evident, from the cases which we have related, that imprisonment, whether of the associate or the separate character, aggravates, as a rule, the morbid condition of mind, and fails altogether of its proper effects. Dr. Maurice Corr suggests the establishment of an institution, intervening in character between a prison and an asylum, and conducted on the principles of a lunatic asylum. An institution so constituted, he conceives (referring particularly to Ireland), would offer the following advantages:—

"I. It would relieve pressure on the Central Asylum, by providing certain moral and medical tests for convicts who *appear to be deranged,* as to the *reality* of their symptoms, whether of idiocy, lunacy, or insanity; and thus, with the most feasible prospect of detecting *malingerers,* it would be sure to produce fair results in doubtful cases.

"II. It would secure the best chance of recovery in hopeful cases.

"III. It would afford ample opportunity for the application of reformatory treatment to all of those classes."

We entirely concur in the suggestion of Dr. Corr, and we believe that an institution of the character he mentions is as much required in England as in Ireland. The lunatic hospitals in London do not supply the need, and in the provinces the great county and the borough hospitals cannot be made available for doubtful cases of lunacy. The projected erection of a state lunatic asylum affords an admirable opportunity for the formation in connexion with it, either as a portion of that building or as a separate building, of a prison for the reception of criminals of doubtful sanity. Such a prison, duly regulated, would become a state hospital for cases of incipient or suspected lunacy among offenders; and its establishment would not only secure the advantages mentioned by Dr. Corr, but it would also put an end to those unsatisfactory and painful instances of frequent change from prison to asylum, from asylum to prison, and from prison to prison, shown in the cases, recorded by Mr. Bradley, of W. G., W. W., and R. D.

